# BEYOND THE DYAD: A SCOPING REVIEW OF CORE COMPONENTS OF FAMILY-CENTERED CAREGIVING INTERVENTIONS

**DOI:** 10.1093/geroni/igae098.0855

**Published:** 2024-12-31

**Authors:** Kelly O’Sullivan, Alexandra Jackson, Sooyoun Park, Raven Weaver

**Affiliations:** Washington State University, Pullman, Washington, United States; Pacific University, Forest Grove, Oregon, United States; Washington State University, Pullman, Washington, United States; Washington State University, Pullman, Washington, United States

## Abstract

Dementia prevalence is predicted to increase alongside the number of older adults, magnifying the demand for informal caregiving. While there are many effective caregiver interventions for people living with dementia and their care partners, most interventions focus on a single caregiver, overlooking the value of including other relatives in the caregiving process. Having education programs that expand beyond the traditional care dyad is crucial in meeting the needs of informal caregivers, especially considering ethnically and racially diverse populations whose cultures center around family. To explore these needs, we conducted a scoping review of 13 previously identified family-centered dementia caregiving interventions to 1) identify core components of these interventions, 2) how effectiveness was measured, and 3) the implementation and impact across cultural groups. We defined family-centered interventions as programs with at least two caregivers participating in the intervention. We initially identified 108 articles, and excluded 41 articles that were either not empirical studies or the intervention did not include at least two caregivers, yielding 67 articles for data extraction. Despite being family-based programs, interventions focused on the care dyad, offering only a few activities to engage multiple caregivers. Similarly, measures of effectiveness were at the individual level (e.g., caregiver burden or depression). However, many of these interventions were tailored and evaluated in multiple cultural groups. The identification of core components can assist with understanding active ingredients, serving as a foundation for programs that can be efficiently tailored to serve diverse families that rely on care involving multiple caregivers.

